# Sparganosis due to *Spirometra* sp. (cestoda; Diphyllobothriidae) in captive meerkats (*Suricata suricatta*)

**DOI:** 10.1016/j.ijppaw.2020.10.005

**Published:** 2020-10-18

**Authors:** Brittany McHale, R. Trey Callahan, Kelsey L. Paras, Martha Weber, Lisa Kimbrell, Yanet Velázquez-Jiménez, Rita McManamon, Elizabeth W. Howerth, Guilherme G. Verocai

**Affiliations:** a Department of Pathology, College of Veterinary Medicine, University of Georgia. 501 D.W. Brooks Drive, Athens, GA 30602, USA; b Department of Infectious Diseases, College of Veterinary Medicine, University of Georgia. 501 D.W. Brooks Drive, Athens, GA 30602, USA; c Riverbanks Zoo & Garden, 500 Wildlife Parkway, Columbia, SC 29210, USA; d Universidad Autónoma del Estado de Puebla. Calle 21 Sur 1103, Barrio Santiago, 72410, Puebla, Puebla, Mexico; e Department of Veterinary Pathobiology, College of Veterinary Medicine and Biomedical Sciences, Texas A&M University, College Station, TX 77843, USA

**Keywords:** Broad tapeworm, Diphyllobothriidea, Herpestidae, Larval cestodosis, Plerocercoid, Sparganosis, Zoonosis

## Abstract

We report three cases of sparganosis due to plerocercoids of the tapeworm *Spirometra* sp. in captive meerkats (*Suricata suricatta*) from a zoo exhibit in the southeastern United States. Two meerkats were euthanized, one due to an uncontrollable seizure and the other due to trauma, and at necropsy cysts containing cestode larvae were observed. A third meerkat had a subcutaneous nodule surgically removed, which contained similar larvae. The third animal died years later, and had numerous cestode larvae in the pleural and peritoneal cavities. The larvae were morphologically identified as plerocercoids of diphyllobothriidean cestodes. On necropsy, multiple nodules, ranging in size from 2.5 to 3.0 cm, were observed in the subcutaneous tissue and muscles. Multifocally, separating skeletal muscle fibers were longitudinal and transversal sections of cestode larva. Histologically, parasitic cysts contained large numbers of neutrophils and macrophages, admixed with proteinaceous material. Molecular and phylogenetic analyses confirmed that specimens from one of the meerkats belonged to the genus *Spirometra* and was closely related to *Spirometra* plerocercoids isolated from a snake from the United States and wild felids from South America. Meerkats likely became infected by ingesting infected second intermediate hosts, such as amphibians and reptiles that may have entered the exhibit. Management practices that minimize access of meerkats and other susceptible hosts to intermediate hosts should be implemented.

## Highlights

• This is the first report of sparganosis in meerkats.
• Parasite identification was based on integrated classical and molecular approaches.
• We highlight the importance of prevention against parasitic infections in zoos.

## Graphical abstract

**Figure:**
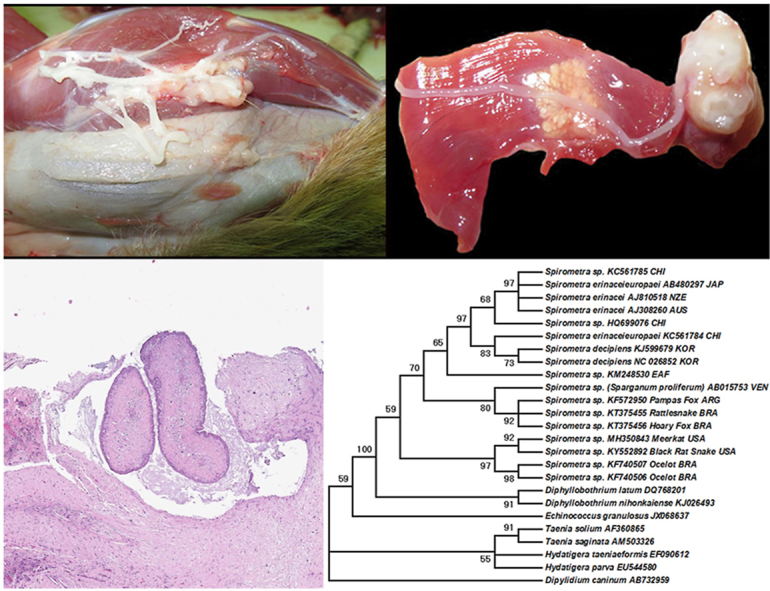

## 1 Introduction

The members of the genus *Spirometra* (Cestoda; Diphyllobothriidea) are intestinal cestodes of carnivore hosts. In North America, *Spirometra mansonoides* (Mueller, 1935), infects domestic cats and dogs, and wild canids, felids and procyonids (Bowman et al., 2002; Mcintosh, 1937; Mueller, 1935, 1974). The validity of this species, however, has been recently questioned by Kuchta et al. (2020), who proposed to provisionally refer to North American isolates as *Spirometra decipiens* species complex 2. Similar to all cestodes, *Spirometra* species have an indirect life-cycle. The hermaphroditic adult cestodes are present in the small intestine of the carnivore definitive host and release operculated eggs. These eggs embryonate in fresh water, in which a ciliated coracidium (first larval stage) emerges. The coracidium then infects copepod crustaceans, mainly of the genus *Cyclops*, in which a procercoid (second larval stage) develops. When infected crustaceans are ingested by the second intermediate host (e.g., amphibians, reptiles, small mammals) a plerocercoid larva develops in the tissues and will serve as the infective stage for carnivore definitive hosts (Mueller, 1938, 1974). In the second intermediate or paratenic hosts, plerocercoids will establish in various organs and tissues, where they may in rare cases even proliferate, causing significant pathology in associated tissues and organs. This condition is known as sparganosis, which, depending on the affected organ and degree of proliferation, may range from benign to fatal. Humans may also be incidental hosts; however zoonotic records of sparganosis in North America are relatively rare with approximately 70 reported cases (Griffin et al., 1996; Kuchta et al., 2015; Mueller et al., 1963; Taylor, 1976). Even more rarely, there has been reports of humans harboring adult *Spirometra* in the small intestine, and therefore, acting as definitive hosts (Kuchta et al., 2020).

We report three cases of infection by *Spirometra* plerocercoids in captive meerkats (*Suricata suricatta*) from a zoological collection from the southeastern United States, confirmed by molecular techniques.

## 2 Materials and methods

### 2.1 Cases

Three captive bred meerkats kept in a zoological collection located at Columbia, South Carolina, southeastern United States entered the collection at seven months of age in 2011, and have been kept in an outdoor enclosure with dirt and sand substrate, with rocks and logs for climbing. All three meerkats were captive-born at the same location in the state of Kansas, United States. Their diet consisted of dry feline commercial diet, various whole prey items, fruits and vegetables, and they were also known to hunt frogs and small mammals. Two meerkats were euthanized due clinical signs referable to the central nervous system, and a third underwent surgery for a removal of a subcutaneous nodule.

**Fig. 1:**
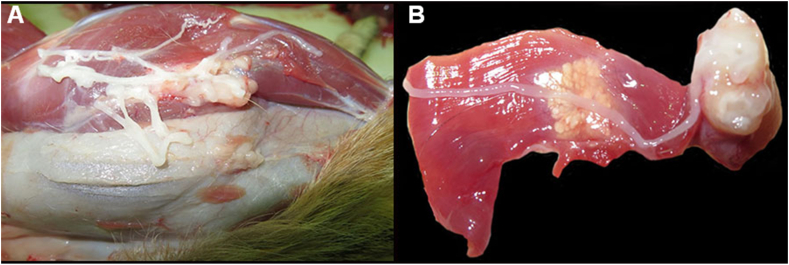
A - Multiple, tan, firm, nodules, ranging in size from 2.5 to 3.0 cm, were observed in the subcutaneous tissue and muscles. B - On incision the large nodules, contained viable cestode larvae, 13.5–18.0 cm long.

The first meerkat was a 4-year-old male (1.12 kg on May 2016) with a history of depression and progressive neurologic signs, including blindness, circling, and tetraparesis that responded to treatment including prednisolone, azithromycin, doxycycline and famotidine. Approximately 3 weeks after discontinuation of treatment the animal experienced uncontrollable seizures and was euthanized. The animal was necropsied and had too numerous to count nodular cysts, 2.5–3 cm diameter, containing viable cestode larvae, 13.5–18.0 cm long, in the subcutaneous tissue and muscles of the abdomen and lateral thorax and separating gluteus superficialis, sartorius, and gracilis muscles of both legs (Fig. 1A and B). Additional findings included dilated cardiomyopathy.

The second meerkat was a 6-year-old male (1.2 kg on June 2017) found in the exhibit with spinal cord trauma. A necropsy was performed at the zoo and formalin fixed tissues submitted to histopathology. Numerous larval cestodes (plerocercoids) were observed grossly in the thoracic cavity, including some free floating in the pleural space, attached to the pleural wall, as well as embedded in the mediastinum. This animal was being treated for dilated cardiomyopathy and was heartworm antigen positive.

The third animal was a 6-year-old male (1.075 kg on June 2020) that was found depressed with a large bruise and swelling in the inguinal region, which at surgery was found to be a large area of subcutaneous inflammation and granulation tissue containing free cestodes. This animal was ultimately euthanized. On necropsy the clinician found larval cestodes in the pleural cavity, peritoneal cavity, a cyst containing a plerocercoid in the right kidney. Additionally, there was a walled off plerocercoid in the left inguinal area, and in the muscle of the left hamstring.

Sections of tissues containing parasites were fixed in 10% neutral buffered formalin, embedded in paraffin, sectioned, and stained with hematoxylin and eosin (H&E) for histological examination.

Intact and fragments of the larvae from the cysts were consistent with plerocercoids of diphyllobothriidean cestodes. Specimens were cleared in lactophenol and examined under a compound microscope. The use of diagnostic samples for research was covered by an institutional animal care and use protocol (AUP # A2017 05–014).

### 2.2 Molecular analysis

Fragments of plerocercoids recovered from meerkat 1 were processed for genomic DNA extraction manually using the Qiagen DNeasy Blood & Tissue kit (Qiagen, Germantown, MD, USA) following the manufacturer's protocol for tissue. The only modification was that parasite fragments were incubated for 12 h in a dry heat block.

Polymerase chain reaction (PCR) was performed targeting a fragment of the cytochrome *c* oxidase subunit 1 (cox1) a gene of the mitochondrial DNA (mtDNA) following a modification of a previously published protocol (Bowles et al., 1992). Each 25 μL reaction included: Go™Taq Green Master Mix (Promega, Madison, WI, USA), 0.5 μmol/L of each primer (forward: 5′-TTTTTTGGGCATCCTGAGGTTTAT-3′ and reverse: 5′-TAAAGAAAGAACATAATGAAAATG-3′), and 2 μL of DNA template. Cycling parameters consisted of an initial denaturation at 94 °C for 5 min, followed by 35 cycles of 94 °C for 30 s, 52 °C for 1 min, and 72 °C for 1 min, with a final 7 min extension at 72 °C, as per Hoggard et al. (2019). PCR product was purified using the E. Z.N.A. Cycle Pure kit (Omega Bio-Tek, Norcross, GA, USA). Sanger sequencing was performed in both directions using BigDye Terminator Cycle Sequencing (Applied Biosystems).

### 2.3 Phylogenetic analysis

The generated fragments of the cox1 gene were edited and aligned by Clustal W in MEGA X (Kumar et al., 2018). Phylogenetic analysis was performed in MEGA X using the Maximum Likelihood method with 1000 bootstrap replicates. The best fit nucleotide substitution model for the data set was HKY + G. Homologous sequences of other *Spirometra* isolates. Outgroups were selected following Waeschenbach et al. (2017).

## 3 Results

Based on integrated morphology, gross and histopathological findings, and molecular methods, the larval cestodes infecting the three meerkats were identified as *Spirometra* sp. Morphology consistent with a plerocercoid was supported by their ribbon-like, unsegmented structure, and some fragments containing typical slit-like scolices.

**Fig. 2:**
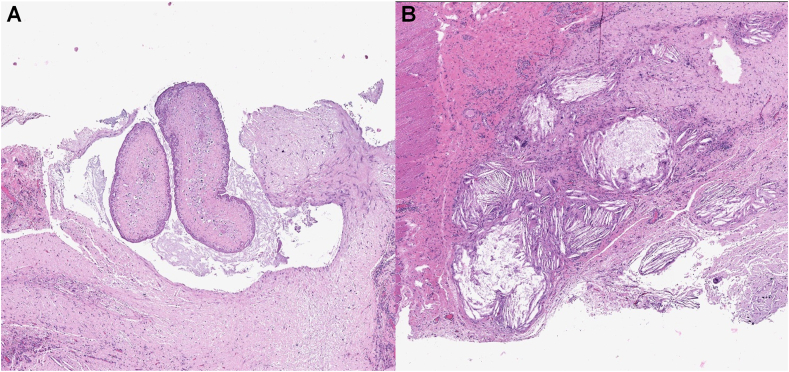
A - Multifocally, separating skeletal muscle fibers are longitudinal and transversal sections of cestode larvae. The larvae form a parasitic cyst. B - Inflammation associated with the larvae consists of macrophages, multinucleated giant cells, and acicular clefts (cholesterol clefts).

Histologically, plerocercoids were present in skeletal muscles (Fig. 2A), subcutis, diaphragm and perirenal tissue and were characterized by a fibrous capsule variably infiltrated with lymphocytes and plasma cells and fewer macrophages and contained larval cestodes and sometimes degenerate neutrophils and proteinaceous debris. The larvae had a thin 4–5 μm wide, eosinophilic tegument, fibrillary eosinophilic parenchyma and scattered calcareous corpuscles (Fig. 2B).

**Fig. 3:**
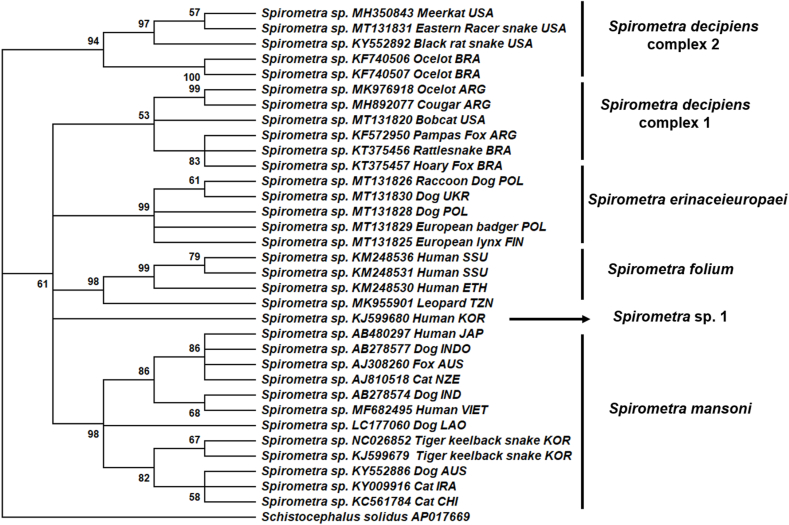
Maximum Likelihood phylogenetic tree depicting the relationships of the *Spirometra* isolate from a captive meerkat (Case 1; MH350843) from the United States, and isolates of *Spirometra* from various hosts worldwide based on a fragment of the cytochrome *c* subunit 1 gene. Branches with less than 50% bootstrap support were collapsed. Bootstrap support shown besides branches are based on 1000 replicates. When available, common name of animal host and geographic origin are included following GenBank accession number. ARG = Argentina, AUS = Australia, BRA = Brazil, CHI = China, ETH = Ethiopia, FIN = Finland, IND = India, INDO= Indonesia, IRA = Iran, JAP = Japan, KOR = South Korea, NZE = New Zealand, POL = Poland, SSU = South Sudan, TZN = Tanzania, UKR = Ukraine, USA = United States of America, VEN = Venezuela, VIET= Vietnam.

The fragment of the cox1 gene (409bp; MH350843) was successfully amplified, confirming the morphological and histopathological identification of plerocercoids of *Spirometra* sp. BLAST search showed that our sequences was most similar (99.5%, 407/409bp) to *Spirometra* isolates of plerocercoid found in a black rat snake (KY552892), and an eastern racer snake (MT131831) from the United States. These likely belong to the same species, within the *S. decipiens* species complex 2 proposed by Kuchta et al. (2020), with strong support (97% bootstrap support; BS). These were comprised within a well-supported clade (94% BS) along isolates from Brazilian ocelot (*Leopardus pardalis*) (Fig. 3), despite their relatively lower maximum identity (88–89%). All other *Spirometra* sequences were included in a clade with moderate support (61% BS). Within this clade sequences included in *Spirometra erinaceieuropaei* from Europe (99% BS), *Spirometra folium* from Western Africa (98% BS), and *Spirometra mansoni* from Asia and Oceania had strong support (99% BS) (Fig. 3). *Spirometra* sp. 1 *sensu* Kuchta et al. (2020) was also included within this major clade.

The clade comprised by sequences belonging to *Spirometra decipiens* species complex 1 was not strongly support (53% BS), and included isolates from wild felids, canids, and a reptile intermediate host from both South and North America, including a bobcat (*Lynx rufus*) from the United States.

## 4 Discussion

To our knowledge, these are the first reported cases of sparganosis in captive meerkats. Our diagnosis was confirmed by integrated gross and histopathological, parasitological, and molecular findings. Despite the fact the meerkat has not been previously reported as paratenic hosts for *Spirometra* sp., sparganosis have been reported from a wide variety of vertebrate hosts, including amphibians, reptiles, bids, and mammals, including humans (Bengtson and Rogers, 2001; Kondzior et al., 2018; Kuchta et al., 2020; Oda et al., 2016; Zhang et al., 2020). The varied clinical presentations and different outcomes of the reported cases may be associated with the location and intensity of infection by plerocercoids of *Spirometra* and co-morbidities. Nevertheless, sparganosis can lead to fatal outcomes in various vertebrate groups, especially in its proliferative form (Berger et al., 2009; Buergelt et al., 1984; Drake et al., 2008; Kikuchi and Maruyama, 2020; Miyadera et al., 2001).

Given the meerkats were born and raised in captivity, these cases are likely autochthonous to the United States. Until recently, all previous reports from North America are generally assumed to be *S. mansonoides*, but now should be provisionally allocated into the *S. decipiens* species complex 2 (*sensu* Kuchta et al., 2020). Various domestic and wild carnivores have been reported infected by adult *Spirometra* sp. in the eastern United States, and in other areas of North America. In fact,. infection by *Spirometra* is relatively commonly found in cats (Hoggard et al., 2019; Mueller, 1935; Wyrosdick et al., 2017), and less commonly in dogs (Conboy, 2009; Nagamori et al., 2020). Among wildlife, there have been reports of *Spirometra* sp. infecting bobcats (*Lynx rufus*) (Harkema and Miller, 1964; Heidt et al., 1988; Kuchta et al., 2020), cougar (*Puma concolor*) (Foster et al., 2006), raccoons (*Procyon lotor*) (Schaffer et al., 1981), coyotes (*Canis latrans*) (Gompper et al., 2003), and gray foxes (*Urocyon cinereoargenteus*) (Conti, 1984). Generally, these carnivore definitive hosts harbor adult tapeworms in their small intestine, and infections are assumed to have little or no clinical importance. However, there are reports of dogs and cats also serving as intermediate hosts, presenting with proliferative sparganosis. In these cases, plerocercoids were found superficially in a dog (e.g., subcutaneous tissues and intermuscular fascia) and in the serosa of viscera in a cat (e.g., liver, spleen, and stomach) (Buergelt et al., 1984; Drake et al., 2008), both resembling the necropsy findings of the meerkats of this report.

Historically, *S. mansonoides* was assumed to be the only species occurring in North and South America (Mueller, 1974; Mueller et al., 1975), and it infects a variety of definitive and intermediate hosts. Recent molecular evidence, however, may support the existence of multiple species associated with wild and domestic species in the Americas (Almeida et al., 2016; Petrigh et al., 2015), and it has been proposed to refer to such isolates as *S. decipiens* species complex, comprised by two species complexes, each composed by at least two species (Kuchta et al., 2020). For instance, our phylogenetic analysis suggests that the North American isolates and the Brazilian ocelot isolates (Almeida et al., 2016) are closely related, but likely not conspecific. Interestingly, however, the only sequence available from a bobcat from the United States clusters within *S. decipiens* species complex 1, and therefore a distinct species from the meerkat isolate. This highlights the complex historical biogeography and the existence of multiple cryptic species of *Spirometra* in the New World. Moreover, theBrazilian ocelot isolates seem to be distantly related to those from other wild South American felids (ocelot and cougar from Argentina), and canids, including the Pampa's fox (Lycalopex gymnocercus) from Argentina and the hoary fox (*Lycalopex vetulus*) from Brazil (Almeida et al., 2016; Petrigh et al., 2015; Scioscia et al., 2014).

Additional neotropical carnivores have been reported to as hosts for *Spirometra*, including at least three canids, namely as the Darwin's fox (*Pseudalopex fulvipes*) (Jiménez et al., 2012), the crab-eating fox (*Cerdocyon thous*), and the maned wolf (*Chrysocyon brachyurus*) (Curi et al., 2010); and six felids, namely the jaguar (*Panthera onca*), the margay (*Leopardus wiedii*), the kodkod (*Leopardus guigna*), the southern tiger cat (*Leopardus guttulus*), the jaguarundi (Herpailurus yagoauroundi), and the cougar (Acuña-Olea et al., 2020; Arrabal et al., 2020; Vieira et al., 2008). Molecular data, especially with matching morphological data, remains scarce for *Spirometra* isolates across the Americas. Therefore, the molecular characterization of isolates from different hosts and geographic regions in the Americas and worldwide will shed light into the true, hidden biodiversity of the genus (Scholz et al., 2019) and would be informative for investigating potential reservoir hosts for sparganosis in animals and humans.

Sparganosis may be acquired through two different transmission routes: i) when a second intermediate/paratenic host ingests an infected crustacean copepod first intermediate host containing a procercoid; or ii) when a vertebrate host, which is not a suitable definitive host, ingests a second intermediate hosts containing plerocercoids, with plerocercoids establishing in this new host (Mueller, 1974). It is more likely that the meerkats ingested an amphibian, reptile or rodent second intermediate hosts that entered their enclosure. Our findings suggest the presence of infected amphibians or reptiles in the premises of the Zoo, which may have become infected by ingesting freshwater crustaceans present in surrounding water features or outside the meerkats’ enclosure. Therefore, it is plausible that infected definitive hosts may have access to certain areas of the Zoo or are present in the surrounding areas. According to zoo personnel, feral cats are present in the premises of the park, and these are likely the reservoir host for *Spirometra*. Alternatively, among the potential wild carnivore hosts, raccoons are more likely to establish within or in the surroundings of zoological parks in search for food and may be another potential source of environmental contamination *Spirometra* eggs. Zoological parks should implement strategies to mitigate the potential transfer of pathogens from feral cats and other wild carnivores to captive animals. These prevention strategies should take into consideration the complex life history of different pathogens, including *Spirometra*, as many of these may use small rodents, birds, reptiles and amphibians as intermediate or paratenic hosts, which may be preyed upon by the captive carnivore or omnivore species.

## 5 Conclusions

Meerkats may serve as paratenic hosts for tapeworms of the genus *Spirometra*, and infections by its plerocercoids may be potentially fatal.

## Declaration of competing interest

The authors declare that they have no known competing financial interests or personal relationships that could have appeared to influence the work reported in this paper.
